# Electrochemical Solid-State Electrolyte Reactors: Configurations, Applications, and Future Prospects

**DOI:** 10.1007/s40820-025-01824-y

**Published:** 2025-06-23

**Authors:** Weisong Li, Yanjie Zhai, Shanhe Gong, Yingying Zhou, Qing Xia, Jie Wu, Xiao Zhang

**Affiliations:** 1 Department of Mechanical Engineering, The Hong Kong Polytechnic University, Hung Hom, Kowloon, 999077 Hong Kong, SAR People’s Republic of China; 2 Research Institute for Smart Energy, The Hong Kong Polytechnic University, Hung Hom, Kowloon, 999077 Hong Kong, SAR People’s Republic of China; 3 Research Centre for Carbon-Strategic Catalysis, The Hong Kong Polytechnic University, Hung Hom, Kowloon, 999077 Hong Kong, SAR People’s Republic of China; 4 Shenzhen Research Institute, The Hong Kong Polytechnic University, Shenzhen, Guangdong People’s Republic of China; 5 PolyU-Daya Bay Technology and Innovation Research Institute, Huizhou, Guangdong People’s Republic of China

**Keywords:** Solid-state electrolyte reactors, Electrolyzer design and optimization, Electrochemical synthesis, Electrochemical carbon capture

## Abstract

The advancement of clean electricity is positioning electrochemical reactors at the forefront of future electrosynthesis technologies. Solid-state electrolyte (SSE) reactors emerge for their distinctive configurations and ability to produce high-purity fuels and chemicals efficiently without additional purification steps. This marks a substantial development in electrochemical synthesis. In this perspective, we critically examine cutting-edge innovations in SSE devices with particular emphasis on the architectural introduction of core cell components, novel electrochemical cell configurations, and assembly methodologies. The use of SSE reactors is presently undergoing a pivotal transition from fundamental laboratory investigations to large-scale engineering implementations, demonstrating remarkable progress in multiple domains: (1) sustainable synthesis of high-value organic acids (formic and acetic acids), (2) production of critical oxidizers hydrogen peroxide (H_2_O_2_) and liquid fuels (ethanol), (3) ammonia (NH_3_) production, (4) carbon capture technologies, (5) lithium recovery and recycling, and (6) tandem or coupling strategies for high-value-added products. Importantly, the transformative potential in environmental remediation, particularly for airborne pollutant sequestration and advanced wastewater purification, is addressed. Additionally, the innovative architectural blueprints for next-generation SSE stack are presented, aiming to establish a comprehensive framework to guide the transition from laboratory-scale innovation to industrial-scale deployment of SSE devices in the foreseeable future.

## Highlights

- Solid-state electrolyte reactors are elucidated in terms of their distinctive electrochemical architecture to facilitate the efficient direct synthesis of fuels and chemicals without the need for traditional purification steps.
- The core components, variable configurations, and distinct electrochemical reaction mechanisms of different chambers are systematically summarized.
- Potential future application scenarios and advanced cell stack designs are pointed out.

## Graphical abstract

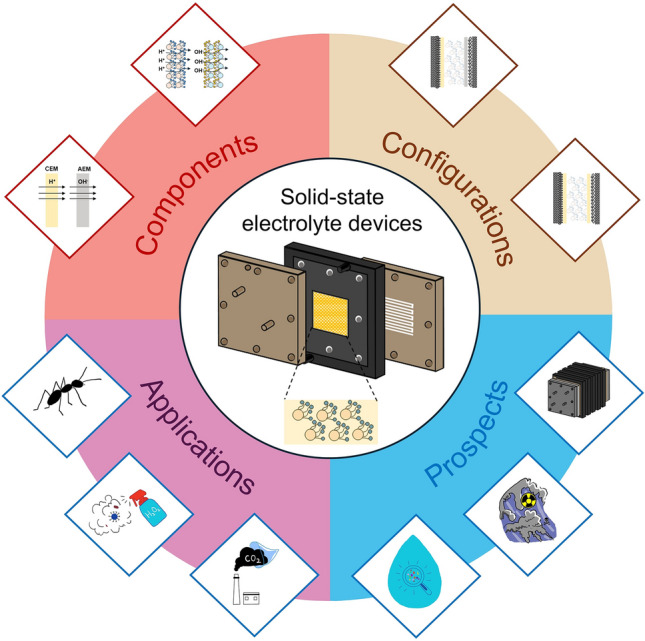

## Introduction

Energy conversion and utilization refer to the transformation of energy from different forms to meet various application requirements [1]. Recently, with the rising global energy demand and environmental concerns, efficient and sustainable energy conversion technologies have become increasingly attractive. Among these, reactors play a crucial role in converting and utilizing renewable energy [2]. Electrochemical reactors, powered by electrical energy, stand out for their high efficiency, controllability, and eco-friendliness. They convert renewable energy sources (like solar and wind energy) into chemical energy, which can be utilized for valuable chemical synthesis, energy storage, and the remediation of environmental pollutants, all while reducing greenhouse gas emissions. With advancements in catalysis and materials science, the performance of electrochemical reactors has been enhanced, facilitating efficient energy conversion and downstream applications. Typically, the efficiency of the energy conversion process is largely dependent on two factors: the electrocatalyst and the electrochemical reactor. While considerable progress has been made in the design and synthesis of electrocatalysts, the engineering and construction of electrochemical reactors are equally essential for energy utilization. Currently, recent electrosynthesis efforts predominantly emphasize enhancing the performance of electrocatalysts, often overlooking the design and optimization of electrochemical reactors [3–5]. Therefore, advancing and stable electrochemical reactors are essential for realizing the full potential of electrochemical synthesis. For example, the revolution of electrochemical reactors has greatly accelerated the progress of water splitting [6] and carbon dioxide (CO_2_) reduction [7] from laboratory scale to practical applications. This development involving the electrochemical reactors upgraded from H-type cells to flow cells and membrane electrode assembly (MEA) cells has fostered a steady step of electrosynthesis toward high efficiency, stability, and energy saving.

Solid-state electrolyte (SSE) reactor has emerged and gained rapid traction in recent years. By using solid electrolytes instead of traditional liquid electrolytes, the SSE reactor elevates the safety and durability of the reactor, while also being easily scalable and mobile for different applications. Another notable feature of the SSE reactor is the utilization of two ion exchange membranes and a distinctive SSE chamber. This design allows the ion recombination process, a key step in electrochemical synthesis, within the buffer chamber. This setup prevents the mixing of products, reactants, or electrolytes, allowing for the production of high-concentration and high-purity chemicals. With these advantages, SSE reactors are now widely employed for the production of various high-purity valuable chemicals/fuels, such as hydrogen peroxide (H_2_O_2_) [8], formic acid [9], ethanol [10], ammonia (NH_3_) [11], captured CO_2_ [12], lithium recovery and recycling [13, 14], and integrated strategies for the synthesis of C_2+_ molecules, such as acetic acid [15–18], ethylene glycol (EG) [19, 20], and glucose [21].

**Fig. 1:**
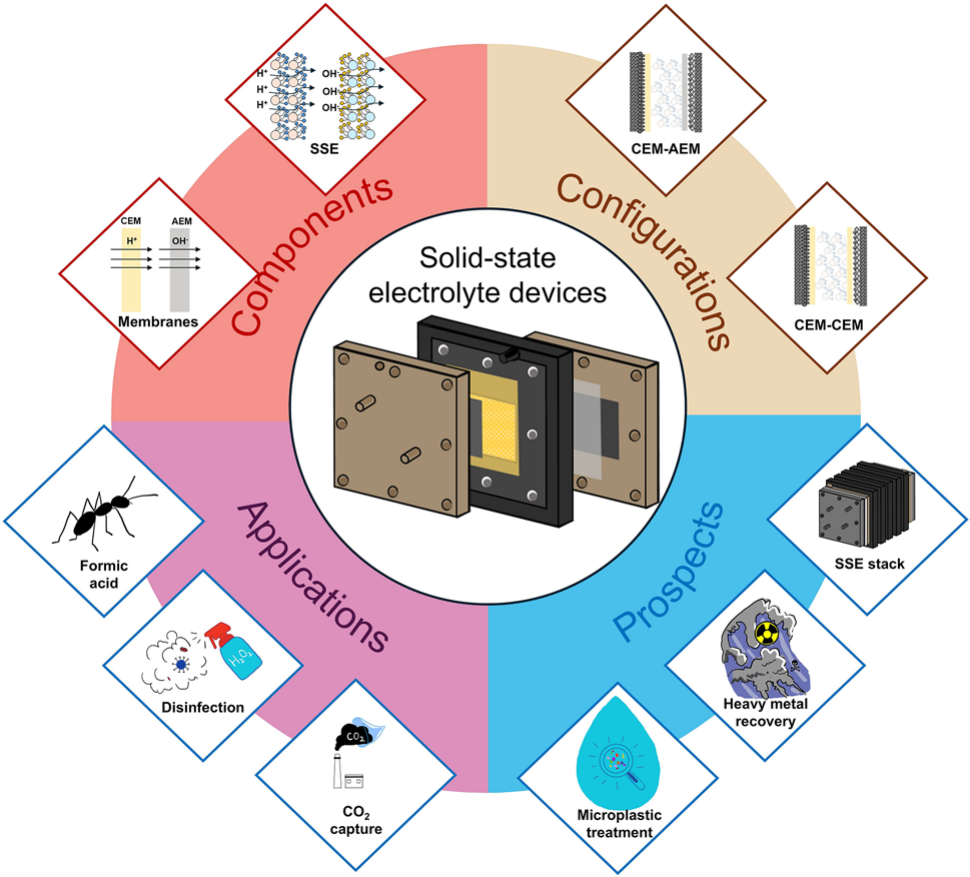
Structure configurations and applications of SSE devices. This diagram summarizes the core components and variable configurations of the SSE reactor, which allow for a variety of application scenarios such as production of formic acid and H_2_O_2_, CO_2_ capture, as well as possible future applications in microplastic treatment, and heavy metal recycling

Distinct from conventional review articles that predominantly focus on material-level electrocatalyst development, this perspective provides a unique vantage point by delivering a systematic deconstruction of SSE reactors through hierarchical analysis of their core components, operational architectures, and variable configurations. We first introduce the development history of electrochemical reactors and compare SSE devices with traditional electrochemical reactors, highlighting the innovative structures and properties of SSE devices. Followed by the introduction and elaboration of construction components, we provide detailed design and selection ideas for developing SSE devices. Different configurations of SSE reactors and the corresponding reactions occurring in cathode, anode, and solid electrolyte chambers (middle chambers) are summarized. Finally, we propose potential applications for SSE devices based on existing designs and applications, with potential to expand the range of applications in microplastic processing and heavy metal recovery. We particularly emphasize the underexplored engineering paradigms governing stack-level integration, where modular cell configurations and multiphase transport dynamics collectively determine practical performance ceilings. By establishing structure–property–application relationships across laminating structure, this work could bridge the application gaps between fundamental electrochemical reactors and deployable device technologies (Fig. 1).

## Development of Electrochemical Reactors

Electrochemical reactors have an abundant development history and undergo several stages of evolution. Their development is largely driven by their applications, with typical examples like the production of hydrogen (H_2_) and oxygen (O_2_) by water splitting, synthesis of H_2_O_2_ from O_2_, formic acid and acetic acid through CO_2_ electrochemical reduction reaction (CO_2_RR), etc. For water splitting, it can be traced back to 1800 when J. Ritter first realized a complete water electrolysis in a voltaic pile [22]. Then after a-century development, the industrial application of water splitting had been achieved in 1902 by alkaline electrolyzers [23]. Until now, water splitting reactors have been upgraded to electrolyzer stacks and remain the mainstay of hydrogen production. For H_2_O_2_ production, a trickle-bed electrochemical reactor used for H_2_O_2_ electrosynthesis emerged as a pioneering model for electrochemical reactors in chemical synthesis in 1979 [24]. Consequently, the evolution from H_2_O_2_ fuel cell [25] to flow type [26] and solid polymer fuel cell [27] enabled the electrosynthesis relative to higher concentrations of H_2_O_2_. The introduction of the flow cell reactor in 2017 marked a progress in producing high concentrations of H_2_O_2_ [28]. By 2019, the development of a SSE reactor allowed for the electrosynthesis of H_2_O_2_ with high concentration and purity [8]. Likewise, CO_2_RR was initially realized in a H-type cell in 2001 [29]. Subsequent improvements in Faraday efficiency and current density were achieved in flow cells [30, 31] and MEA cells [32, 33]. This progress facilitated the electrochemical synthesis of a variety of products, such as carbon monoxide (CO) [34], formic acid [35], acetic acid [36], ethylene [37], and other multi-carbon products [9, 35, 38]. Notably, the utilization of SSE devices directly prompted the CO_2_RR, enabling chemical production with high concentration and purity.

**Fig. 2:**
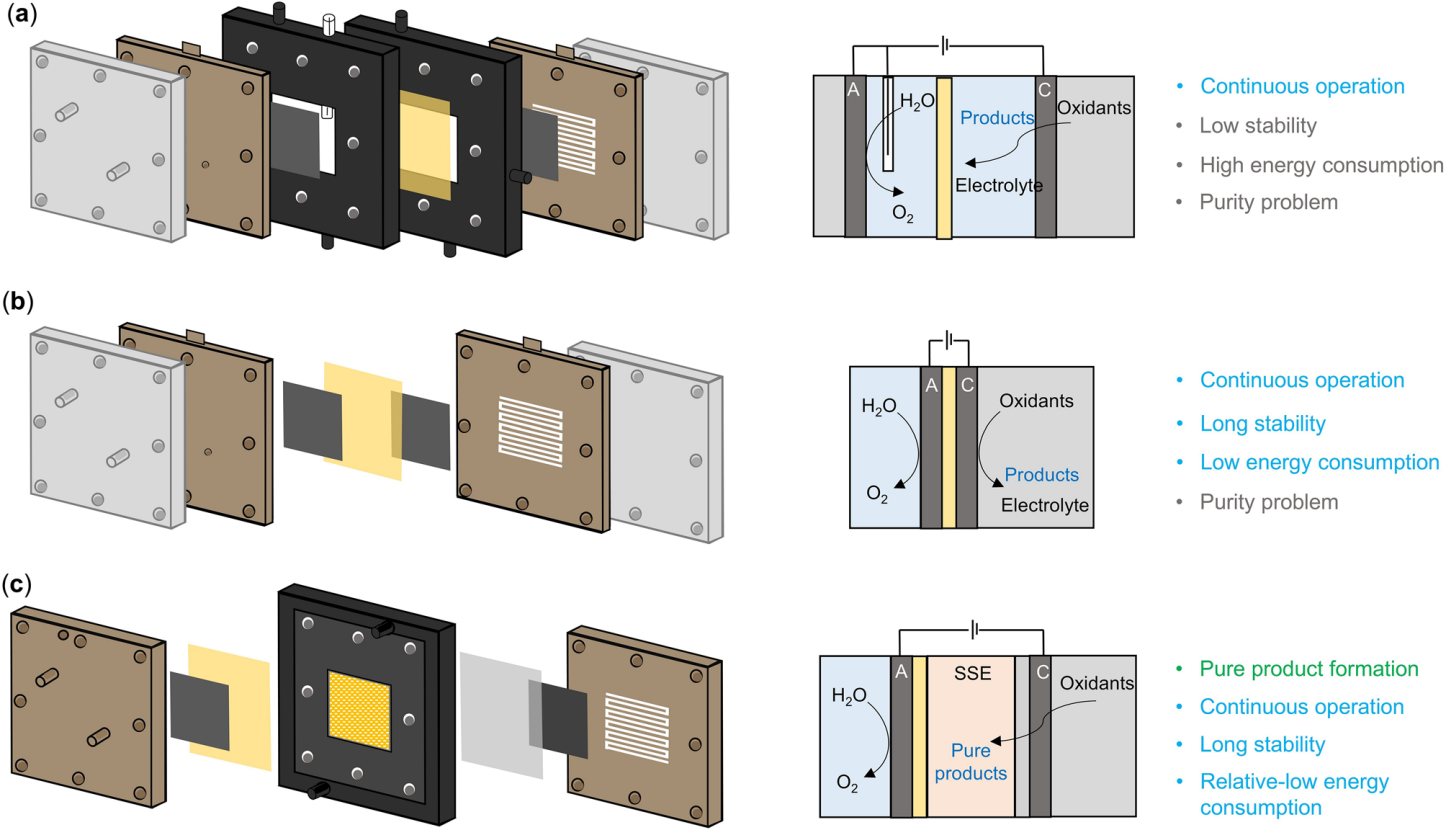
Cell structures of different electrochemical reactors. **a** A common flow cell, composed of two liquid chambers, one membrane, one reference electrode (white cylinder), two metal plates, and two end plates. **b** A typical MEA cell, consisting of two metal plates (served as liquid chambers), one membrane, and two end plates. **c** A typical SSE reactor, including two metal plates (functionalized as liquid chambers and end plates), two membranes, and one middle chamber (stuffed with solid electrolytes for ion recombination and product separation). The anode and cathode sides are denoted as A and C, respectively

The early explorations of electrochemical reactions began with H-type cell, but it is unsuitable for large-scale continuous production owing to constraints related to reactor size, the low solubility of gaseous reactants, and elevated electrical resistance. With the advent of flow cells, electrochemical reactors advanced to the device level. A typical flow cell is depicted in Fig. 2a, in which oxidation reactions occur at the anode and reduction reactions happen at the cathode. The anodic reactions, such as oxygen evolution reaction (OER), water oxidation reaction (WOR), or hydrogen oxidation reaction (HOR), typically generate protons, which then penetrate the cation/proton exchange membrane (CEM/PEM). Simultaneously, the gaseous reactants are pumped across the backside of cathode catalyst to arrive at the reaction site. Then, a reduction reaction occurs at the cathode to generate anions, which combine with the anode-produced protons to form the target products. Inevitably, the target products, particularly the liquid products, tend to intermingle with the catholyte, leading to a reduction in purity and consequently incurring relatively higher downstream purification costs.

In addition, due to the direct contact between liquid electrolytes and catalysts, water flooding commonly happens on the cathode catalysts, seriously affecting the stability of the flow cell and hindering large-scale application. In contrast, a MEA reactor, which integrates the gas channels and cathode chamber into a fully symmetrical sandwich-like structure, significantly minimizes the ohmic losses associated with liquid electrolytes [39]. As a result, energy consumption and stability are thus improved (Fig. 2b), while the target liquid product still mixes with electrolytes. Thus, further purification steps are necessary which increase the total cost for the whole production process and limit the large-scale development of these reactors for electrochemical production.

The SSE reactor, which combines features of flow cells and MEA cells, has recently emerged as a promising technology [8, 9, 40, 41]. Different with traditional MEA reactor, the SSE device has an additional middle chamber between cathode and anode, filled with solid electrolytes instead of liquid solution (Fig. 2c). The solid electrolyte is a type of porous ion exchange resin that guarantees high ion conductivity during electrolysis. In addition, the middle chamber filled with solid electrolyte allows for easy removal of target products by flushing with deionized water or inert gas, enabling the production of high-purity, high-concentration products. Additionally, the use of solid electrolytes can mitigate water flooding issues common in flow cells by preventing direct liquid contact with catalysts. Therefore, the use of solid electrolytes not only reduces the resistance of the entire cell (compared with liquid electrolytes), but also modifies the problem of water flooding, contributing to reducing energy consumption and dramatically improving stability of SSE reactors.

While SSE devices show attractive advantages, they also face challenges in reaction efficiency, product selectivity, and device durability [9, 42, 43]. For instance, although the middle solid electrolyte layer reduces the cell resistance, its cathode-to-anode distance is still larger than zero-gap MEA cells, leading to increased energy consumption. In addition, the complicated structure, due to the extra middle layer and membranes, results in a more intricate assembly process and lower durability compared to MEA cells. These issues have sparked interest in the design and optimization of reactors. This includes strategies such as reducing the thickness of middle chambers to lower cell resistance [12], employing flexible and stable CEM-CEM structures to enhance durability and applicability [11, 44, 45], and replacing traditional CEM membranes with custom ion-selective membranes to achieve specific separation applications [13]. In the coming years, electrochemical reactors are anticipated to assume an increasingly pivotal role in sectors such as renewable energy, environmental protection, and green chemistry. As technology continues to advance and costs progressively decrease, the commercialization and industrial application of electrochemical reactors will be further facilitated, bolstering global efforts toward sustainable development. Electrochemical reactors are not only an important part of the modern electrochemical industry, but also one of the key technologies to realize energy transition and environmental protection. The interdisciplinary cooperation and innovation will be an important driving force for the development of electrochemical reactors.

## Structure Configurations of SSE Devices

### Structure of SSE Devices

**Fig. 3:**
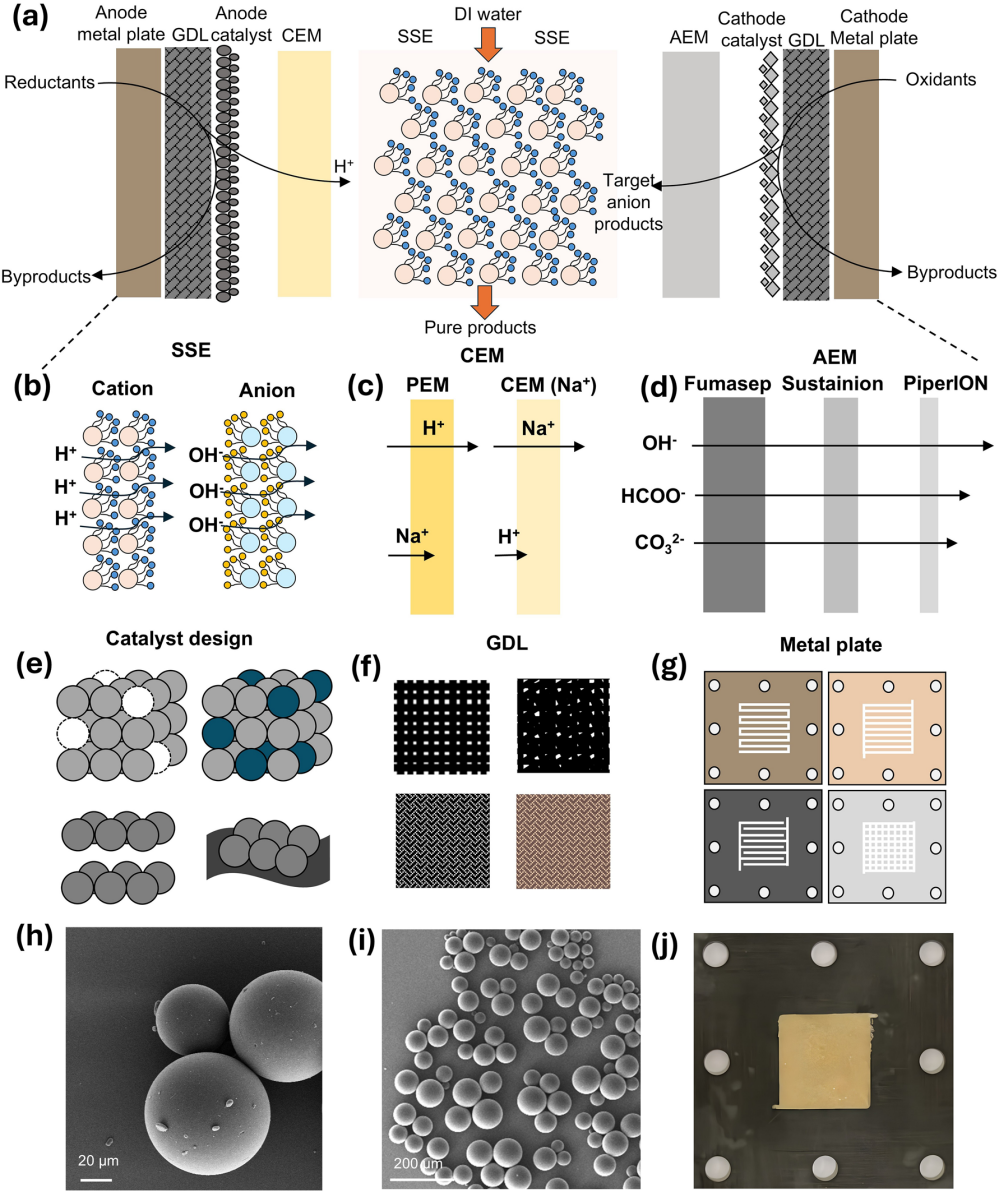
Structures and components of SSE devices. **a** A cross-section illustration of an SSE device, including membranes, catalysts, GDLs, and metal plates. **b** Structures and types of SSE, containing anionic and cationic solid-state electrolytes for different ion transport. **c** Different types of cationic membranes, such as PEM and CEM (Na^+^) according to the selectivity requirements of the ions. **d** Different types of anionic membranes with different thicknesses and functional groups, therefore the transport rates of anions with different valences and molecular volume vary. **e** Common structure of catalyst involves vacancy, alloying, layered, and supported types. **f** Commonly used GDLs include carbon cloth, carbon paper, carbon felts and titanium felts. **g** Metal plates commonly used in SSE devices include titanium alloy plates with different contents, graphite plates and stainless steel plates, and flow channels including serpentine, parallel, interdigitated, and chessboard types. The SEM images of real SSE particles with **h** 20 μm and **i** 200 μm. **j** Picture of stuffed SSE in middle chamber

**Fig. 4:**
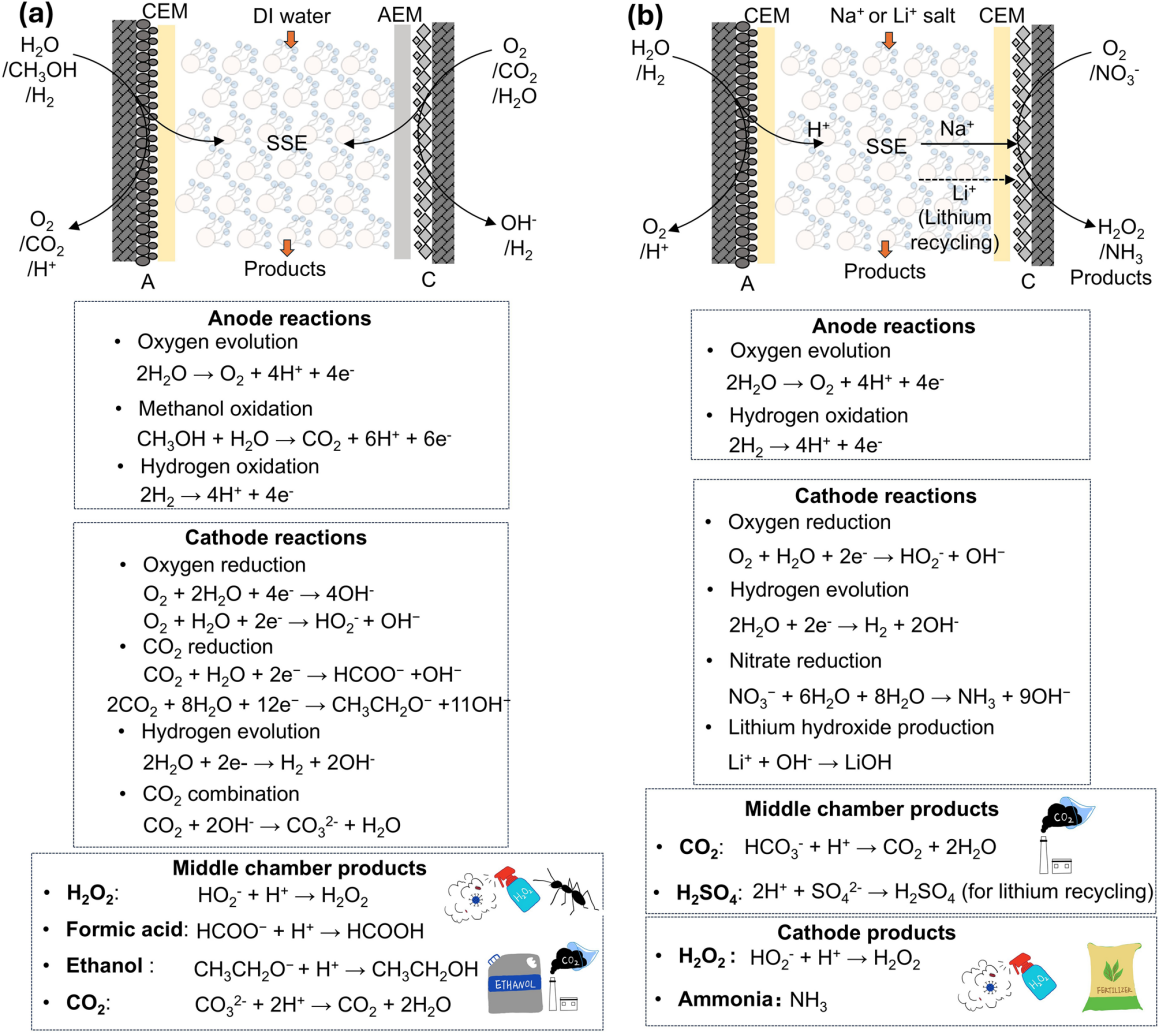
Various configurations and relevant reactions in SSE devices. a A CEM-AEM configuration. Utilizing this design, the middle chamber provides room for ionic recombination, which facilitates generating pure products including H_2_O_2_, formic acid, ethanol, and CO_2_. **b** A CEM-CEM configuration. In this system, the products are not confined to the middle chamber. The function of the CEM at the cathode enhances ion shielding from H^+^ to Na^+^ /Li^+^ , boosting the production of NH_3_ from NO_3_RR, H_2_O_2_ or lithium hydroxide (LiOH). A dashed line indicates the transport of Li^+^ ions from the middle to the cathode chamber during the lithium recovery process

Generally, the SSE device typically possesses a symmetrical structure consisting of two ion exchange membranes, two catalyst layers, two gas diffusion layers (GDL), two metal plates, and one middle solid-state electrolyte chamber (Fig. 3a). The entire cell is composed of a symmetrical structure, and the component that distinguishes it from other electrochemical reactors is the membrane-encapsulated solid electrolytes. There are mainly two types of configurations reported in the literature, including CEM-AEM and CEM-CEM structures. The CEM-AEM structure refers to the use of a cation membrane in the anode side and an anion membrane in the cathode side within the SSE reactor, enabling the selective migration of cations and anions (Figs. 3a and 4a). Under the external electric field, the oxidation reaction happens at the anode catalyst layer, which generates electrons and hydrogen ions (protons). These protons then pass through the CEM into the middle chamber, driven by the external electric field and concentration difference. Meanwhile, the reduction reaction takes place at the cathode catalyst layer and the reactants obtain electrons and undergo a reduction process to produce the anionic form of the target product, which passes through the AEM and enters the middle layer under the impetus of the electric field and the concentration difference. Then the anions and protons in the middle layer will meet and combine to form the target products, which constitute the ionic flow circuit. The electrons generated by the anode return to the power supply, while the cathode consumes the electrons provided by the power supply, forming a circuit of electron flow.

Differently, a CEM-CEM configuration denotes the use of two cation membranes within the SSE reactor, facilitating the migration of cations and achieving specific effects (Fig. 4b). The CEM-CEM configuration runs a completely different ion flow, where the electrons generated by the reaction of the anodic catalyst layer enter the middle chamber, and the anions generated by the cathodic catalyst layer are not able to enter the SSE layer due to the repulsion of the CEM. The ion flow that occurs in the middle chamber is a complementation and exchange of ions, for example, the electrochemical release of CO_2_ from the work by our group [45]. And the reduction of nitrate in the work of Wang and Sibani et al. [11] both utilized the exchange of sodium ions and hydrogen ions in the SSE chamber. The ion flow formed at this space constitutes an ion cycling loop. It is worth noting that the recombination reaction of ions in the SSE chamber is a chemical process, which does not involve electron transfer. Therefore, the ionic reaction rate is related to the ionic transfer rate, which is closely linked to the ionic conductivity of solid electrolytes [46, 47]. While the oxidation and reduction reactions occurring at the electrodes are electrochemical reactions in addition to the rate of ion conduction at the membrane interface, which is also related to the rate of electron transfer, and the rate of mass transfer.

### Design and Selection of Core Components

The core component of SSE device is the solid-state electrolytes, which is a class of functionalized ion exchange resins. Among them, the cation exchange resin is generally functionalized with sulfonic acid groups on the branched chain [48–51], and the protons are transported and moved through the negatively charged sulfonic acid groups (Fig. 3b). The anion exchange group is generally functionalized with quaternary amine groups, and such positively charged groups can play a role in the transport of anions [52–54]. The size of SSE particles is typically between 50 and 500 nm, where larger sizes (smaller mesh size) result in higher cell resistance, while smaller sizes (larger mesh size) result in lower mobility of the products generated therein. Therefore, the selection of particle size for SSE is a trade-off between cell resistance and product yield.

Different types of cationic membranes are illustrated in Fig. 3c, where PEM membranes mainly carry out protons but small amounts of cations such as sodium ions can also be transported, whereas CEMs (e.g., Na-type) selectively transmit sodium ions, shielding most of the protons from penetration. The branch groups of the PEMs are similar to that of the cationic solid-state electrolytes, which are functionalized by sulfonic acid groups for proton transfer [55, 56]. In contrast, CEMs with sodium ion selectivity are designed with a tailored pore size based on the high hydration radius of sodium ions, which allows selective separation and crossover of sodium ions [57, 58]. Compared to cationic membranes, anion exchange membranes are much more diverse, and commonly used models include Fumasep [59], Sustainion [60], and PiperION [61]. Due to their thickness and surface functional groups, their transfer rates for anions of different valences and sizes are generally as: OH^−^ > HCOO^−^ > CO_3_^2−^ (Fig. 3d) [62, 63]. The thickness of both anionic and cationic membranes shows a positive correlation with cell resistance. The stability of thinner membranes has been an important issue in the development of anionic membranes. Additionally, AEM plays a crucial role in isolating the solid-state electrolyte from the gaseous reactants at the cathode. Localized strong acidic and alkaline environments, pressure difference due to gas molecules may impose challenges on the strength and durability of the AEM. Therefore, the choice of appropriate thickness of membranes is also a matter of detailed consideration of cell energy consumption and stability.

The design and structure of catalysts directly influence the selectivity and stability of SSE devices. Catalyst design is tailored to specific target products, such as CO_2_RR and oxygen reduction reaction (ORR). Common structures for cathodic electrocatalysts typically include vacancy-type, alloy-type, layered, and supported configurations (Fig. 3e). The structural diversity and tunability of cathodic catalysts offer a rich design space for electrochemical applications, enabling precise control over catalytic properties through tailored atomic configurations and compositional engineering. This versatility establishes catalyst design as a pivotal research frontier in electrocatalysis, where systematic manipulation of active sites and interfacial environments can unlock enhanced performance metrics [64–66]. As for anode catalysts, their general choice is relatively fixed. Since most current applications use anode electrodes as counter electrodes, the efficient and stable OER catalyst: Iridium dioxide (IrO_2_) is usually the most conventional choice [67–69].

In SSE devices, the catalysts employed are not merely simple catalyst sheets, rather, they are supported on GDL substrates that feature gas diffusion channels [70, 71]. These substrates must possess sufficient electrical conductivity, porous structures, and stable mechanical strength (Fig. 3f). Commonly used GDL materials include carbon cloth, carbon paper, carbon felt, and titanium felt, with thickness typically arranged in the order of: carbon felt > carbon cloth > titanium felt > carbon paper. In terms of pore size, the order is: carbon cloth > carbon felt > titanium felt > carbon paper. The thinnest carbon paper results in the lowest electrical resistance within the SSE reactor, while its long-term stability under rapid gas and liquid flow conditions is inferior to that of GDLs mentioned earlier. Therefore, the selection of GDLs must consider a comprehensive balance between mass transfer, electrical resistance, and durability issues.

The SSE reactor is secured with metallic end plates, unlike traditional flow cell and MEA cell, it integrates the flow channel plates of the gas or liquid chambers with the end plates, facilitating easier cell assembly. The materials used for the metallic end plates typically differ between the anode and cathode, wherein anode requires oxidation-resistant and acid-corrosion-resistant titanium alloy materials [72, 73], while the cathode can utilize graphite plates or stainless steel plates [74]. The flow channels etched into the metal plates play a crucial role in the transport of reactants and the removal of products. The design and selection of these flow channels are inspired by fuel cell configurations, including serpentine, parallel, interdigitated, and pin (chessboard) types (Fig. 3g) [75–77]. In current applications, serpentine flow channels are the most common due to their stable flow characteristics and relatively uniform concentration distribution. In contrast, chessboard and parallel flow channel designs can accommodate higher flow rates, while interdigital configurations facilitate more uniformly distributed mass flow. In practical design, the selection of flow channels determines the efficiency of mass transfer, with the rapid supply of reactants and the swift removal of products being indispensable steps in electrochemical synthesis.

The middle plate is the core component of the entire SSE reactor. This chamber is filled with SSE particles. The scanning electron microscope (SEM) diagrams are shown in Fig. 3h, i. The middle plate is generally made of polyoxymethylene (POM) material, an insulation and corrosion-resistant material, which has an open and penetrating window, and the solid electrolyte particles need to be filled tightly and flatly inside the punch (Fig. 3j). The thickness of the middle plate and the flatness of the SSE filling (the thickness of the SSE layer) also contribute to the overall resistance of the cell. Therefore, appropriately adjusting the thickness can further help reduce the energy consumption of the device [12]. The area of the SSE window should be consistent with the flow channel area of the metal plates, otherwise, discrepancies may arise in ion transport (membrane transport) and ion recombination (SSE surface). In practice, the size of the SSE window and the area of the flow channel can be adjusted according to the desired target yield to accommodate electrosynthesis at high current densities. In summary, the components that constitute the SSE device are intricately linked to its yield, performance, stability, and energy consumption. The design and selection of each component are essential to ensure that the SSE reactor possesses competitive advantages comparable to other types of electrochemical reactors.

## Configurations of SSE Devices

The configurations of SSE devices have evolved to address the varying requirements for anion/cation crossover under different reactions and the production of specific target products. The two common configurations currently available are the CEM-AEM (Fig. 4a) and CEM-CEM (Fig. 4b) types. Although these two configurations exhibit similar symmetrical structures, differing in the type of cathodic membrane employed, the principles they operate on are fundamentally divergent. This differentiation in the underlying mechanisms underscores the complexity of their functionality, suggesting that each configuration harnesses distinct electrochemical processes and transport phenomena [78]. Moreover, their applications and performance characteristics can vary selectively, underscoring the importance of membrane selection.

For the CEM-AEM configuration, the main working electrode is located at the cathode, and a variety of cathodic reduction reactions can be carried out, such as ORR (2-electron reduction to H_2_O_2_ and 4-electron reduction to hydroxide (OH^−^), CO_2_RR production to formic acid, and hydrogen evolution reaction (HER). More specifically, in addition to electrochemical reactions, the alkaline environment generated in the cathode chamber can have the effect of capturing CO_2_, which is shown as CO_2_ recombination in Fig. 4a. The anionic form of these target products will cross the AEM into the SSE layer of the middle chamber by electromigration. In contrast, the anode, as the counter electrode of the electrolytic cell, has a relatively fixed reaction, while the main requirement is a stable supply of protons (H^+^). ORR, methanol oxidation reaction (MOR), and HOR are all optional anode-pair electrode reactions. The H^+^ produced by these anodic reactions also passes through the CEM (generally a PEM) by electromigration process into the SSE layer, which, due to its good ability to conduct protons, encourages them to move until they meet the anions. At this point, the reactions occurring in the middle chamber are all chemical reactions involving non-electron. The generated electrically neutral products are the target products, such as H_2_O_2_ [8], formic acid [9], ethanol [10], or CO_2_ [12]. It is worth noting that the SSE layer is generally wetted by DI water. SSE materials are essentially sulfonated copolymers (Fig. 3b) and proton conduction takes place between the sulfonate groups in the form of hydrated ions [79, 80]. Therefore, maintaining sufficient humidity is a necessary factor for the SSE layer to carry out ionic recombination reactions. The generated target products are generally collected and stored by flowing deionized water to produce a high concentration of product streams. Gaseous products such as formic acid can be purged out of the chamber by pumping inert gases such as nitrogen. Exceptionally, the SSE reactor with AEM-CEM structure can also facilitate tandem or coupling reactions. For instance, the H_2_O_2_ generated in the middle layer can react with ethylene to produce EG [19, 20]. Additionally, the acetic acid produced via carbon monoxide reduction reaction (CORR) can be coupled with a bioreactor to generate a series of multi-carbon molecules [18, 21]. This highlights the significant potential of the SSE reactor for synthesizing high-carbon-chain organic compounds.

For the system of CEM-CEM, the core principle is the modulation induced by cation electromigration. Unlike AEM-CEM, this configuration allows the generation of target products at the cathode, such as H_2_O_2_ or NH_3_ (Fig. 4b). In the electrosynthesis of H_2_O_2_, the cathode employs the same PEM as the anode. Alkali metal cations are introduced into the middle chamber, allowing them to pass through the PEM into the cathode, thereby shielding against protons that may permeate and preventing the decomposition of H_2_O_2_ [44]. For nitrate reduction reaction (NO_3_RR), a sodium ion-selective CEM is used to allow Na^+^ to diffuse across the membrane and shield the protons (leading to HER), promoting selective NH_3_ production at the cathode [11]. Different from CO_2_ capture process in the CEM-AEM configuration, CO_2_ liberation process in the middle chamber can be achieved in this configuration by pumping the solution containing carbonate or bicarbonate directly into the middle layer, wherein protons diffusing from the anode can combine with the carbonate or bicarbonate ions and resulting in the liberation of CO_2_. It can be summarized that the variable configuration of the SSE reactor can be utilized not only to generate pure products by ion recombination in the middle chamber, but also to achieve product synthesis in the cathode chamber using ion shielding or ion isolation. This strategy of decoupling ion recombination and ion shielding/isolation can rapidly expand the application scope of SSE devices. Using a similar CEM-CEM structure, an innovative ion migration strategy has been employed in the SSE reactor, facilitating the dissociation of lithium sulfate within the middle chamber and the recycling of lithium ions at the cathode [14]. Its applications are no longer confined to traditional reduction reactions but hold promise for producing multi-carbon products in CO_2_RR, generating protonated intermediates for organic synthesis, and facilitating the separation of metal ions.

## Utilization of SSE Devices

### Applications of AEM-CEM Configuration

**Fig. 5:**
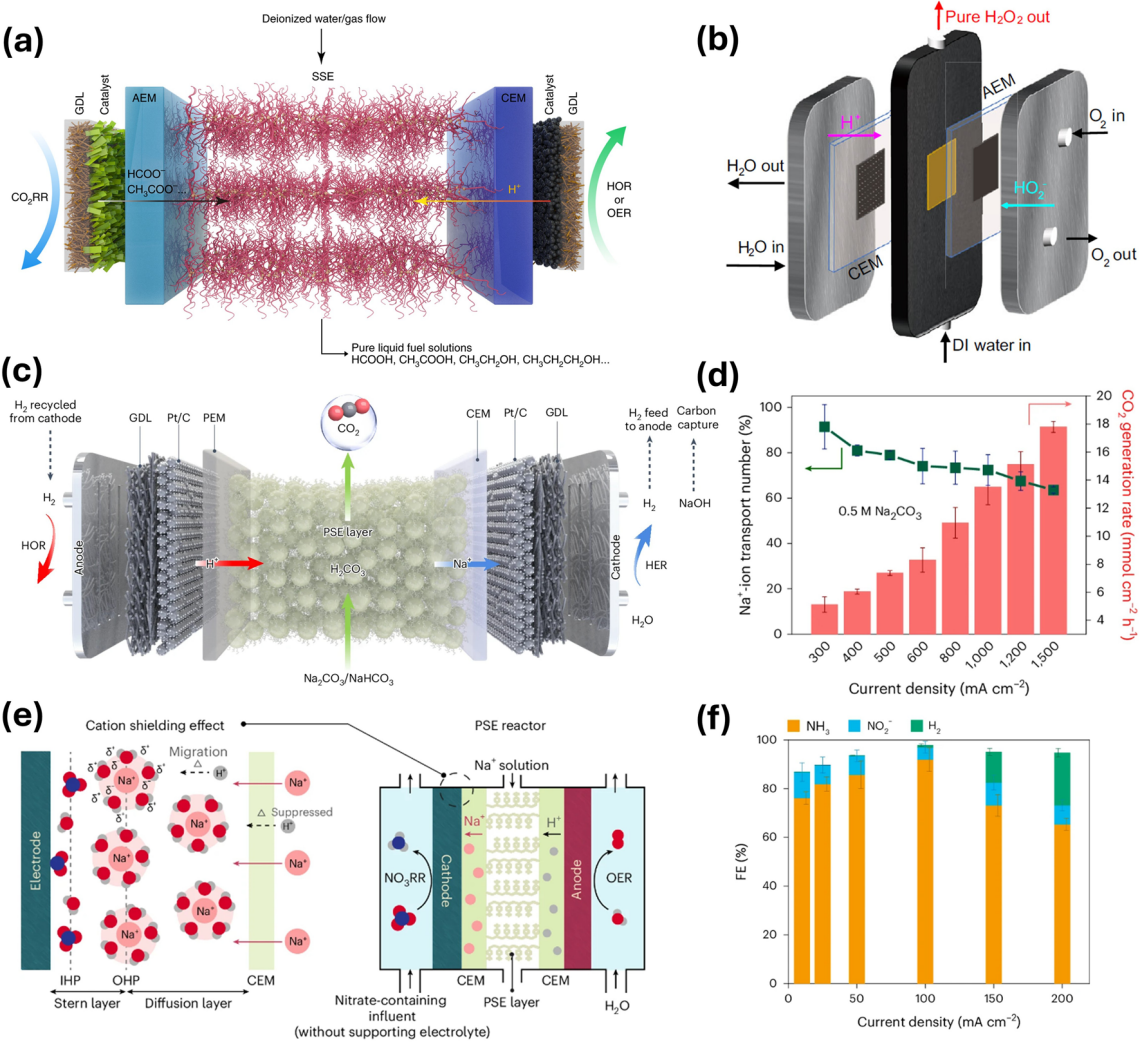
Current applications of SSE devices. The schematic of SSE devices for **a** formic acid production [9], Copyright 2019, Springer Nature; and **b** H_2_O_2_ production [81], Copyright 2021, Springer Nature. **c** Different configurations for electrochemical CO_2_ capture and regeneration in a CEM-CEM system [45], Copyright 2024, Springer Nature. **d** Na^+^ ion transport number and CO_2_ generation rate under different current densities [45], Copyright 2024, Springer Nature. **e** Cell structure for NO_3_RR to NH_3_ production with cation shielding effect induced by Na^+^ ions [11], Copyright 2024, Springer Nature. **f** Faradic efficiencies (FEs) of NH_3_ under different current densities [11], Copyright 2024, Springer Nature

Currently, the limited practical applications of SSE devices can be categorized under two configurations. The representative studies from various applications are specifically highlighted in Fig. 5, showcasing the integration of SSE devices into practical scenarios. Xia et al. achieved the first electrosynthesis of high concentration (20% wt) and high purity H_2_O_2_ in 2019, utilizing the SSE device in a CEM-AEM configuration [8]. In the same year, he and his colleagues also realized the first high concentration (up to 12 M) formic acid production in an SSE reactor and elaborated on its potential use for the production of multi-carbon liquid fuels (e.g., acetic acid, ethanol and propanol, etc.), as shown in Fig. 5a [9]. Zhu et al. achieved acetic acid synthesis with a purity of up to 98% using CORR within this configuration [15]. What is more, Zheng et al. employed a tandem design, first conducting electrochemical CO synthesis, followed by acetic acid production via CORR, and subsequently utilizing a bioreactor for glucose synthesis [21]. This cascade strategy suggests that the SSE reactor has significant potential for the electrosynthesis of long-chain carbon molecules. In 2021, Wang’s group improved and upgraded the catalyst to further increase the selectivity and stability of H_2_O_2_ (Fig. 5b) [81]. In addition to electrochemical reduction for the production of pure products, Kim and his colleagues were keenly aware of the loss of carbon in the CO_2_RR process, where the alkaline environment generated by CO_2_RR captures CO_2_ molecules and converts them into carbonate/bicarbonate forms [82]. On this basis, Zhu et al. systematically investigated the carbon capture process at the cathode and carbon release at the middle chamber using an SSE reactor with a CEM-AEM configuration and realized the capture rate up to 86.7 kg CO_2_ day^−1^ m^−2^. Meanwhile, a two-stage SSE tandem design was deployed to achieve high and low concentration CO_2_ capture, respectively, to maximize capture efficiency [12]. This localized alkalization and acidification design renders electrochemical CO_2_ capture and releases a highly viable method for carbon capture. Furthermore, it is conceivable that the alkaline environment generated by cathodic reactions can absorb a range of acidic gases, such as CO_2_, sulfur dioxide (SO_2_), and nitrogen dioxide (NO_2_). This portable strategy broadens the application scope of SSE devices, making them promising candidates for air pollutant treatment and hazardous gas removal.

### Applications of CEM-CEM Configuration

The practical application of the CEM-CEM configuration was achieved by Willauer et al. in 2011 [83]. They employed double PEMs along with different types of solid-state electrolytes (Table 1) to facilitate the acidification of the seawater in the middle chamber to release CO_2_ in the middle chamber. Zhang et al. further optimized and refined this strategy into the SSE reactor, enabling the continuous production of CO_2_ in the middle chamber (Fig. 5c) [45]. A cation exchange membrane (Na^+^ type) is employed to facilitate the timely migration of Na^+^ ions from the middle chamber to the cathode. During this process, protons migrating from the anode rapidly acidify the middle chamber, allowing CO_2_ to be released from the carbonate. This design has achieved over 90% Na^+^ ion transport efficiency at a current density of 300 mA cm^−2^, and can reach nearly 18 mmol cm^−2^ h^−1^ at an industrial current of 1.5 A (Fig. 5d). Recently, Chen et al. demonstrated the generation of NH_3_ from nitrate in an SSE reactor using a dual-CEM configuration (Fig. 5e) [11]. The cation shielding effect is applied to promote NO_3_RR performance, attributing to a high FE of NH_3_ up to 92% (Fig. 5f). This strategy of regulating cation migration and cation blocking significantly promotes cation concentration-sensitive reactions, such as CO_2_RR and the two-electron ORR for H_2_O_2_ synthesis.

**Table 1:** Summary of components and performance parameters of SSE devices for electrosynthesis

Products	Anode catalysts	Cathode catalysts	SSE	Anode membrane	Cathode membrane	FE (%)	Current density (mA cm^−2^)	Durability (h) (mA cm^−2^)	References
Formic acid	IrO_2_/C	Sn	Amberlit® IR120	Nafion® 212	Sustainion™ X37	~ 30	140	500 (140)	[86]
Formic acid	IrO_2_/C	2D-Bi	SDB sulfonated copolymer	Nafion	PSM (Dioxide Materials)	93.1	32.1	100 (30)	[9]
Formic acid	IrO_2_/C	nBuLi-Bi	SDB sulfonated copolymer	Nafion 115	AEM (Dioxide Materials and Membranes)	~ 97	450	100 (30)	[43]
Formic acid	IrO_2_/C	Bi_2_O_3_	Amberlit® IR120	Nafion® 324	Sustainion®	91.3	200	1000 (200)	[85]
Formic acid	IrO_2_/Ti mesh	Pb_1_Cu	SDB sulfonated copolymer	Nafion 115	AEM (Dioxide Materials and Membranes)	~ 90	1000	180 (100)	[17]
Formic acid	IrO_2_/Ti mesh	Bi_3_S_2_ nanowires	SDB sulfonated copolymer	Nafion 117	Sustainion® X37-50 grade 60	90	50	120 (50)	[87]
Ethanol	IrO_2_/C	Cu_2_OZn	Dowex 50W X8	Nafion 117	PiperIon /Sustainion AEM (Fuel Cell Store)	> 40%	350	180 (250)	[10]
Acetic acid	IrO_2_/C	Cu_2_O nanotubes	Dowex 1 × 4 copolymer	Nafion	PSMIM	30	700	10 (700)	[15]
Acetic acid	IrO_2_/Ti mesh	Cu-CN	SDB sulfonated copolymer	Nafion	AEM (Dioxide Materials and Membrane)	56.5	100	140 (120)	[16]
Acetic acid	IrO_2_/Ti mesh	GB-Cu	SDB sulfonated copolymer	Bipolar membrane (Fuel Cell Store)	AEM (Dioxide Materials and Membranes)	46	323	140 (250)	[21]
Acetic acid	IrO_2_/C	Ag-doped Cu_2_O NCs	SDB sulfonated copolymer	Nafion 117	PSMIM AEM (Dioxide Materials)	~ 55	400	150 (500)	[18]
H_2_O_2_	IrO_2_/C	XC-72	SDB sulfonated copolymer	Nafion 115	AEM (Dioxide Materials and Membranes)	> 90	200	100 (120)	[8]
H_2_O_2_	IrO_2_/C	B-C	SDB sulfonated copolymer	Nafion 117	AEM (Dioxide Materials and Membranes)	~ 90	30	200 (30)	[81]
H_2_O_2_	IrO_2_/C	BP2000	Dowex 50W X8	Nafion 117	N/A	~ 90	50	500 (50)	[44]
H_2_O_2_	Pt-Ir-Black	N–C	Dowex 50W X8	Nafion 115	Sustainion X37-50 Grade RT	> 90	360	50 (389)	[84]
H_2_O_2_	IrO_2_/C	Vulcan XC-72	N/A	N/A	N/A	> 60	100	1000 (100)	[88]
CO_2_	IrO_2_/C	Pt/C	Dowex 50W X8	Nafion 117	Sustainion	> 90	100	72 (100)	[12]
CO_2_	Mixed precious metal oxide/Ti	316L stainless steel	IRA-120	SybronMC-3470	N/A	N/A	100	N/A	[83]
CO_2_	Pt/C	Pt/C	Dowex 50WX8	Nafion 117	Nafion N2100TX	~ 90 (Na^+^ ion transport number)	100	> 100 (100)	[45]
NH_3_	IrO_2_/C	Ru-CuNW	Dowex 50W X8	Nafion 117	Nafion 117	> 90	100	~ 240 (100)	[11]
LiOH	IrO_2_	Pt/C	SDB sulfonated copolymer	Nafion 117	Lithium conductive ceramic membranes (Ohara)	97.5 (cation transference number)	0.025	1 (0.025)	[13]
LiOH	Pt/C	Pt/C	Dowex 50 W X8	Nafion 117	Nafion N2100TX	~ 90 (cation transference number)	100	10 (100)	[14]

**Fig. 6:**
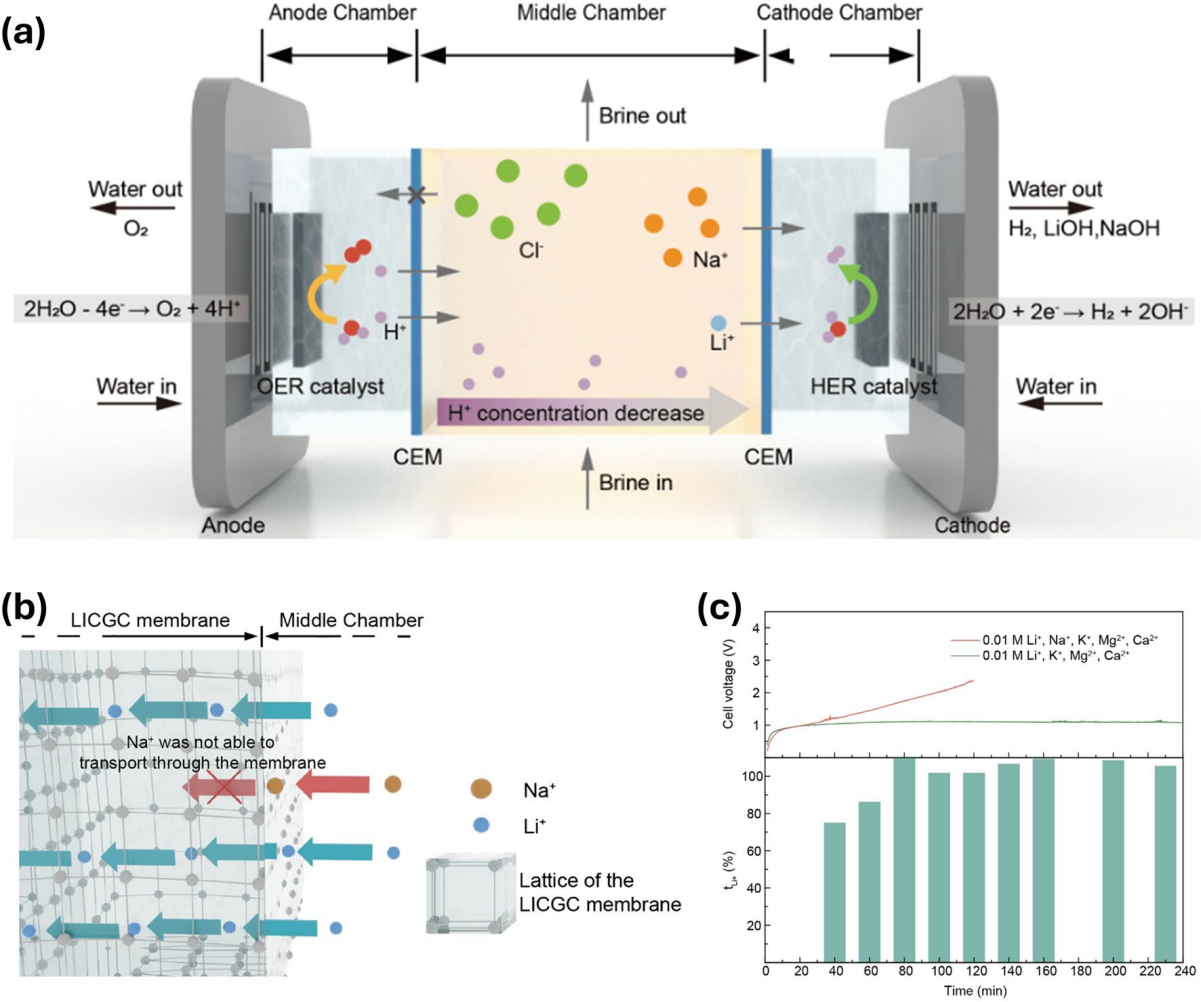
The SSE device with a CEM-CEM configuration for lithium extraction.** a** Scheme of lithium extraction process achieved by the SSE device with a CEM-CEM configuration [13], Copyright 2024, National Academy of Sciences. **b** Li^+^ transfer process on the surface of the LICGC membrane [13], Copyright 2024, National Academy of Sciences. **c** Li^+^ transference number and cell potential for simulated brine (0.01 M Mg^2+^, 0.01 M Ca^2+^,0.01 M K^+^, and 0.01 M Li^+^) [13], Copyright 2024, National Academy of Sciences

Interestingly, in addition to the synthesis of carbon-containing liquid products and H_2_O_2_, the SSE reactor with a CEM-CEM configuration also demonstrates unique performance for Li^+^ ion recovery. Feng et al. successfully employed an innovative ion migration strategy to achieve the separation of brine in the middle chamber and the recovery of lithium at the cathode chamber (Fig. 6a) [13]. The Li^+^ ions in the brine of the SSE chamber will, under the influence of the electric field, pass through the lithium-ion conductive glass ceramic (LICGC) membrane into the cathode, where they react with the OH^−^ produced from the HER to form LiOH, thereby enabling the recovery of Li^+^. Thanks to the customized dimensions of the LICGC membrane, Na^+^ cations in the brine cannot enter the lattice, allowing lithium ions to be effectively separated (Fig. 6b). By simulating the addition of other metal cations (Mg^2+^, Ca^2+^ K^+^ without Na^+^) to mimic complex brine compositions, the dual-CEM design can still achieve over 80% Li^+^ transference number (Fig. 6c). This demonstrates the practicality and effectiveness of this design in practical scenarios. This design significantly broadens the application of SSE devices in the treatment and conversion of cation-containing pollutants in aquatic environments.

Table 1 provides a comprehensive summary of the current applications of SSE devices, detailing the types of SSE, catalysts, CEMs, and AEMs employed. Notably, except for the Pt-Ir-black used by Rawah et al. [84] and the mixed precious metals utilized by Willauer et al. [83] most studies have employed IrO_2_ as a stable and long-term anode catalyst. Given that the cathode serves as the primary site for the generation of product anions, considerable emphasis has been placed on the design and synthesis of cathodic catalysts, which vary widely from carbon materials to transition metals. Although the models of SSEs differ, they predominantly consist of styrenedivinylbenzene (SDB) sulfonated copolymer, providing efficient proton conductivity. CEMs are almost exclusively derived from Dupont's Nafion series, with different models representing varying thicknesses. The use of Nafion membranes on the anode side is similarly aimed at ensuring stable operation of the anode (working electrode). The primary differences in AEMs lie in the anion crossover under various application environments, with the mainstream stable anion membranes being from the Sustainion series.

The performance of SSE devices has reached maturity in the production of formic acid. Yang et al. achieved stable operation of the SSE reactor for up to 1000 h in 2020 [85]. Furthermore, he and his colleagues demonstrated Faradaic efficiencies exceeding 90% while maintaining efficient operation for 500 h as early as 2017 [86]. In the production of acetic acid, typically realized through CORR, the enhancement of stability and selectivity within SSE devices remains a pressing issue. Yan et al. reported operation at a current density of 250 mA cm^−2^ for 140 h, achieving a Faradaic efficiency (FE) of 46% [16]. Long-term stability in the electrosynthesis of H_2_O_2_ continues to be an inherent challenge. Zhang et al. achieved a maximum stability of 500 h (at 50 mA cm⁻^2^) utilizing a CEM-CEM structure in the SSE reactor [44]. In the promising field of electrochemical CO_2_ capture, the stability of SSE reactors has only reached 100 h, indicating a substantial gap from practical industrial applications. Recently, the work involving nitrate reduction to NH_3_ using the SSE reactor has opened new avenues for the application of SSE devices, achieving stability for 240 h at 100 mA cm⁻^2^ [11]. Current applications reveal that SSE reactors still face challenges regarding stability and operation at industrial current densities, necessitating ongoing improvements and design innovations from researchers and technical engineers.

## Prospect of SSE Devices

### Microplastic Treatment and Heavy Metal Recovery

Considering the SSE chamber, this device can be designed for redox reactions of non-electrochemical processes carried out on-site in the middle chamber, which requires an extreme acid-alkaline micro-environment. Since SSE itself consists of polymer resin particles with strong chemical properties, it is promising to design other chemical reactions inside the SSE chamber. For the treatment of water microplastics, it is reported that microplastics can open polymer chains in alkaline conditions by specialized catalysts for degradation [89, 90]. It can be considered that passing wastewater containing microplastics into SSE chamber mixed with catalysts, by utilizing the OH^−^ produced by the cathode and the anionic SSE to conduct OH^−^ to create a localized alkaline environment. Similarly, by utilizing the protons generated at the anode to establish a localized strongly acidic environment in the middle chamber, it may be possible to facilitate certain organic reactions, such as the protonation process in esterification [91, 92], thereby enabling the production of organic esters using the SSE reactor.

In addition, the distinctive configuration of the SSE reactor also allows for the potential recovery of heavy metal ions. For instance, the ion recombination and permeation of heavy metal salts in the middle chamber can lead to the formation of possible acidic stream in the middle chamber and basic heavy metal streams in the cathode side, thereby simultaneously facilitating the recovery of heavy metals and generation of valuable products. Meanwhile, the use of ion-selective membranes designed according to the dimensions of various heavy metal cations also serves as a crucial enabler for the further development of this technology.

### SSE Stack Design

Currently, SSE devices face challenges in meeting practical production demands due to their low operating current density and relatively low output. The design of the electrolyzer stack is expected to be one prospective way to address these issues. A cell stack is a system that combines multiple electrolyzer units in series or parallel to efficiently convert electrical energy into chemical energy [93–96]. It is widely used in water splitting, hydrogen production, and electrochemical synthesis due to their high energy conversion efficiencies, low emissions, and compatibility with renewable energy sources.

**Fig. 7:**
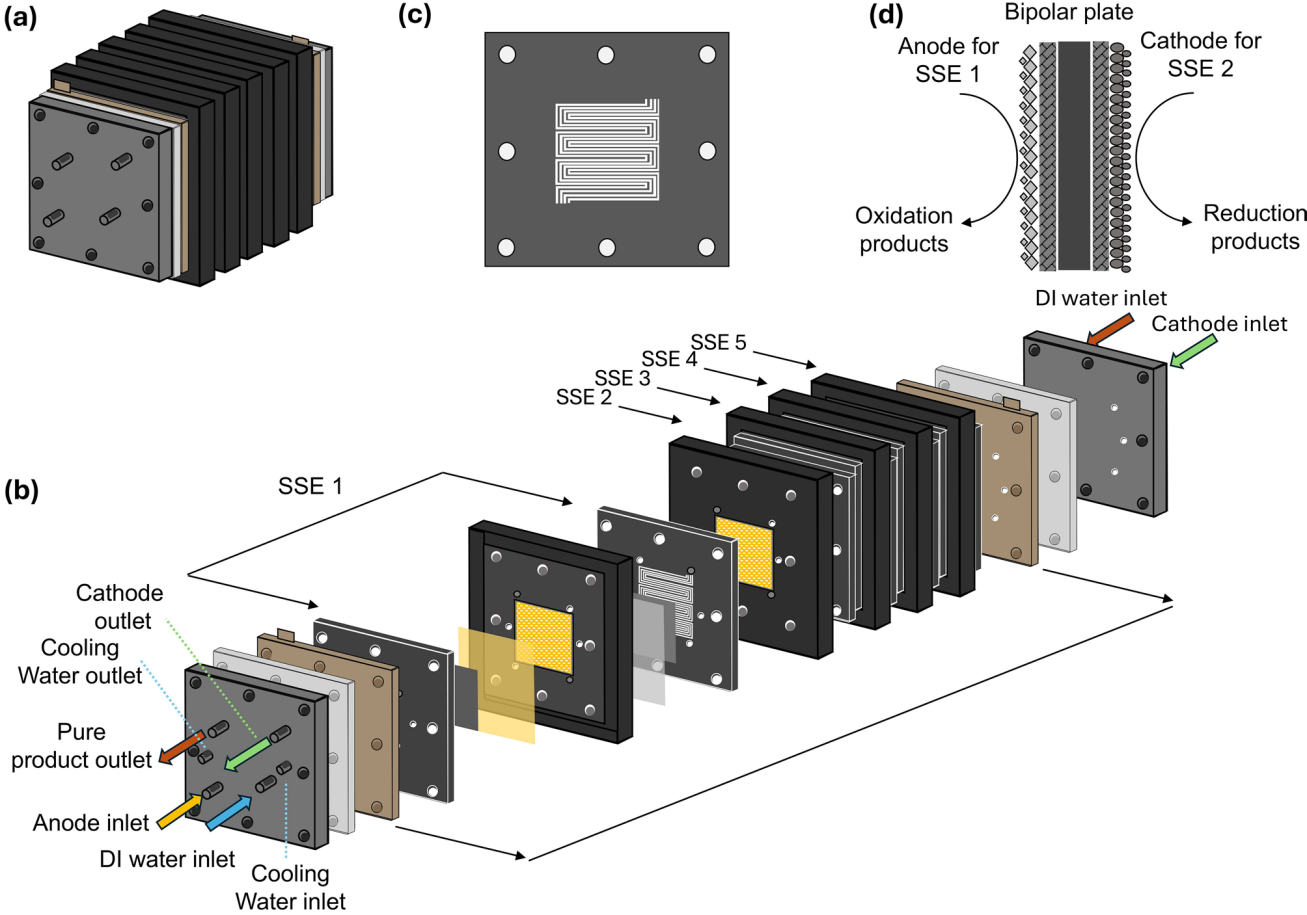
Proposed design and structure of a SSE stack. **a** General appearance of a 5-unit SSE stack. **b** Decomposition view of the stack includes the inlet and outlet piping for both the anode and cathode, and the inlet and outlet for the products in the middle chamber. The assembly consists of compression plates, end plates, current collectors, and five SSE cell units. **c** Upgraded three-channel parallel flow design enhances the flow rate within the stack. **d** Structural design of the bipolar plates. Aside from the end polar plates of the first and fifth cells, each polar plate facilitates the bifunctional properties for oxidation reaction on one side associated with the preceding cell, and the reduction reaction on the opposite side connected to the subsequent cell

The proposed SSE stack draws inspiration from the designs of fuel cell stacks and PEM stacks. Figure 7a illustrates an SSE stack composed of five identical SSE cells. A detailed breakdown of the stack structure is shown in Fig. 7b, which includes compression plates, end plates, current collectors, and the five individual SSE cell units. A three-channel parallel flow design has been proposed to maximize the utilization of the limited electrode plate area (Fig. 7c). By distributing reactants across multiple channels, the pressure drop in a single channel can be significantly reduced, thereby minimizing flow losses and enhancing system energy efficiency. Furthermore, the parallel flow design ensures a more uniform distribution of reactants on the electrode surface, preventing localized concentration polarization and improving the uniformity of current density distribution, which in turn enhances electrolysis efficiency [97]. Additionally, the multi-channel structure facilitates the rapid removal of gas bubbles, reduces the occurrence of gas blockages, lowers interfacial resistance, and improves mass transfer performance.

This innovative stack architecture transcends conventional series or parallel configurations by employing a bipolar plate design, wherein each electrode plate simultaneously operates as the cathode for the preceding electrochemical cell and the anode for the succeeding unit, thereby establishing an integrated and continuous electrochemical pathway (Fig. 7d). Such a developed configuration maximizes the utilization of plate space while minimizing the internal resistance originating from cell thickness. It can be predicted that apart from the improvement in operational efficiency, the SSE stack also contributes to the longevity and stability of the system. By leveraging the bipolar plate configuration, the stack minimizes the number of components required, thereby reducing manufacturing complexity and costs. Additionally, the integration of multiple cells into a single stack enhances the system's scalability, making it adaptable for various industrial applications.

Unlike single SSE devices, the gas and liquid flow channels in the stack require the use of manifolds to distribute the flow streams into each cell unit. This system includes an anode electrolyte stream manifold, a cathode gas stream manifold, and a product stream manifold for the middle chamber. In practical terms, the optimization of the flow distribution within the stack is crucial. The anode electrolyte manifold ensures a uniform supply of the electrolyte to each cell, facilitating ion transport and enhancing reaction kinetics. Similarly, the cathode gas manifold is designed to distribute reactant gases uniformly across the cathode surfaces, promoting efficient electrochemical reactions. The product flow manifold assists in the removal of generated products from the SSE chamber, preventing any potential buildup that could hinder performance. To avoid mixing of cathode and anode as well as product purity issues, each manifold is independent of each other and non-crossing. The arrangement of five laminated units can enhance the overall yield, providing a potential increase of up to five times compared to individual cell operations. Furthermore, this design allows for operation at industrial-level current density with elevated production rates, making it well-suited for large-scale applications.

To optimize the performance of the stack, a thermal management strategy for the five-laminated SSE stack is proposed by incorporating through-plane cooling channels, inspired by the cooling system architecture of PEM stacks (Fig. 7b). Unlike single SSE reactor designs where deionized water flow through middle chamber provides limited thermal regulation while removing reaction products, our integrated cooling circuit-spanning from the first to the fifth cell in continuous circulation addresses the critical thermal challenge arising from the inherently higher impedance (and consequently greater heat generation) of SSE reactors compared to MEA electrolyzers. Furthermore, considering that the membranes in the SSE stack cannot withstand excessively high cell temperatures, issues such as the high-temperature decomposition of middle chamber products arise. It is therefore essential to design a thermal management system, including a cooling circulation loop, to control the system's temperature.

Overall, the design of the SSE stack marks an advancement in electrochemical systems. By integrating established technology principles with cutting-edge structural engineering, the SSE stack offers a solution for an elevated production rate for electrosynthesis. As the global demand for clean energy continues to grow, research and development in electrolyzer stacks are swift, particularly in the context of large-scale fuel and chemical production. This progress underscores a promising potential for the future of sustainable energy solutions.

## Conclusion and Outlook

The emergence of SSE devices implies a new domain in electrosynthesis, fundamentally addressing issues of product purity and eliminating the energy-intensive processes of separation and purification. This advancement holds profound implications for healthcare, liquid fuel synthesis, air and water pollutant treatment. The foundation of this evolution lies in the integration of ion exchange membranes and solid-state electrolytes, resulting in flexible and efficient SSE devices that maintain high efficiency and long stability. These advancements encompass the production of disinfectants (H_2_O_2_), the synthesis of liquid products (formic acid, acetic acid, and ethanol), the technology of electrochemical carbon capture, NH_3_ production from nitrate in wastewater, lithium extraction from brine and tandem or coupling strategies for EG and multi-carbon products production. Cumulatively, these transformative advancements are poised to profoundly reshape the ecological architecture of modern electrochemical synthesis, heralding a paradigm shift toward sustainable green synthesis.

Despite these advancements, the development of SSE devices continues to face a series of challenges. The primary obstacle is the energy consumption issue, which serves as an essential barrier to the widespread adoption of SSE reactors in place of other electrochemical reactors. Achieving this goal requires innovative approaches to membrane materials, GDL materials, electrode plate materials, SSE materials, and their associated thicknesses. Additionally, the development and design of catalysts are crucial for further reducing energy requirements. Another key challenge lies in the mismatch between operational current and yield under industrial application conditions. This necessitates improvements and optimizations in the structure of SSE device, balancing the scaling of reactor size with energy consumption to ensure cost-effectiveness and minimal environmental impact.

Addressing these challenges necessitates a collaborative, interdisciplinary approach that integrates insights from electrocatalytic material design, electrochemical reactor design, fluid dynamics, and reactor thermal management. Ongoing advancements in materials science and reactor manufacturing, coupled with integrated reactor assembly and thermal management strategies, are essential for developing SSE stacks that can adapt to industrial conditions. By perceiving these challenges as stimuli for innovation and progress in electrochemical reactors, we can offer viable solutions to the pressing issues of product purity and concentration in both current and future contexts.
